# The molecular classification ushers in a new era for the treatment of advanced and recurrent endometrial cancer

**DOI:** 10.3389/fonc.2025.1761159

**Published:** 2026-01-12

**Authors:** Liwei Yan, Xiaoxu Ding, Yuanmei Deng, Yachai Li, Xiaoxin Du

**Affiliations:** 1 Department of Gynecology, Hebei University Affiliated Hospital, Baoding, China; 2 Department of Critical Care Medicine, Hebei University Affiliated Hospital, Baoding, China

**Keywords:** advanced and recurrent, endometrial cancer, immunotherapy, molecular classification, surgical treatment, targeted therapy

## Abstract

Endometrial cancer is among the most common malignant tumors of the female reproductive system, with an increasing incidence globally over the past decade and ranking first among gynecological malignancies in developed countries. Although early-stage prognosis is favorable, the mortality rate of advanced and recurrent endometrial cancer remains high, posing a significant clinical challenge. Molecular classification systems, such as TCGA and ProMisE, have identified four distinct molecular subtypes: POLE-mutant (POLEmut), mismatch repair-deficient (MMRd), no specific molecular profile (NSMP), and p53-abnormal (p53abn), each exhibiting significantly different biological behaviors, recurrence patterns, and treatment responses. “Chemotherapy-free” strategies show potential for specific subtypes and offer new avenues for reducing toxicity. Faced with challenges, such as tumor heterogeneity and drug resistance mechanisms, future research should focus on optimizing standardized molecular classification protocols, exploring novel combination therapies, and integrating real-world evidence. A personalized treatment system centered on molecular classification that considers the quality of life and treatment accessibility is crucial for precision medicine in advanced and recurrent endometrial cancer.

## 1 Introduction

**Figure 1:**
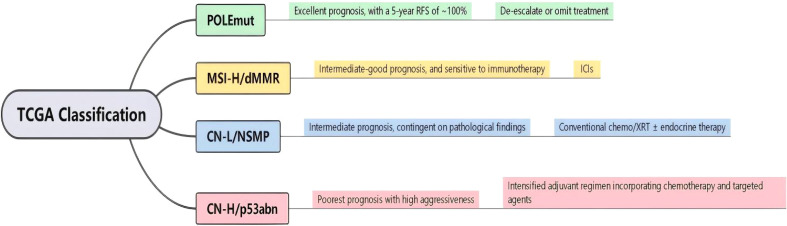
Overview of molecular classification and clinical characteristics of endometrial cancer.

Advanced and recurrent endometrial cancer represents a major challenge in clinical management owing to its high mortality rate (1, 2). Traditionally, endometrial cancer is classified into two types: type I is estrogen-dependent, predominantly comprising low-grade endometrioid carcinoma, accounting for 70–80% of cases, and is associated with a favorable prognosis, whereas type II is non-estrogen-dependent, including serous carcinoma, clear cell carcinoma, and carcinosarcoma, accounting for 20–30% of cases, and is characterized by high aggressiveness, a propensity for early metastasis, and poor prognosis (3–5). Historically, treatment for endometrial cancer has relied on clinicopathological features, such as histological type, FIGO stage, and differentiation grade for risk stratification and regimen selection. However, this approach fails to adequately explain the prognostic differences between patients with similar clinicopathological features (6, 7). To elucidate its biological heterogeneity, the molecular classification system proposed by The Cancer Genome Atlas (TCGA) in 2013 categorizes endometrial cancer into four subtypes: POLE-mutant (POLEmut), microsatellite instability-high (MSI-H), copy-number low (CN-L), and copy-number high (CN-H). The 2023 FIGO staging system integrated molecular classification, embodying an “anatomic-molecular” dual-dimension staging concept for more accurate prognostic assessment and treatment guidance, as different molecular subtypes predict distinct recurrence risks and patterns. POLEmut has the most favorable prognosis owing to its high tumor mutational burden that leads to enhanced immunogenicity. It carries a low risk of recurrence regardless of the clinical stage, with multiple retrospective studies showing that its 5-year recurrence-free survival (RFS) approaches 100%. Consequently, international guidelines recommend close follow-up, rather than adjuvant chemotherapy or radiotherapy, for early-stage patients with POLEmut (8–10). Even after recurrence, the responses to immunotherapy or chemotherapy remain good (11, 12). MSI-H or mismatch repair-deficient (MMRd) tumors are often associated with Lynch syndrome or sporadic mismatch repair deficiency, frequently manifest as distant metastases, and are sensitive to immune checkpoint inhibitors (PD-1/PD-L1 monoclonal antibodies), with objective response rates (ORR) reaching 40–50% (13–15). CN-H (primarily characterized by TP53 mutations) highly overlaps with high-grade histological types, such as serous carcinoma. Even in clinical stage I, the risk of recurrence is significantly higher than in other subtypes, necessitating intensified adjuvant therapy. The NCCN guidelines recommend platinum-based chemotherapy combined with targeted therapy (anti-angiogenic agents) as a priority for patients with advanced-stage p53abn tumors.P53abn recurrence is often platinum-resistant and prone to peritoneal metastasis and multiorgan involvement, conferring the worst prognosis (4, 16, 17). NSMP comprised the largest proportion of patients with recurrence patterns that were intermediate between the other three subtypes. Its treatment strategy still requires integration with clinicopathological features; however, the addition of molecular markers can further refine the stratification (**Figure 1**).

This review aimed to systematically summarize the current treatment landscape for advanced and recurrent endometrial cancer. We focus on integrating recent advances in molecular classification-guided precision therapy, the discrepancies between real-world effectiveness data and clinical trial results, and exploring future therapeutic directions.

## 2 Treatment strategies for advanced and recurrent endometrial cancer

Therapeutic decision-making for advanced and recurrent endometrial cancer requires comprehensive consideration of tumor biology, patient functional status, and accessibility of medical resources (18). Regardless of the need for surgical intervention, combination systemic therapy, including chemotherapy, immunotherapy, and targeted therapy, is necessary, with selective administration of radiotherapy.

### 2.1 Surgical treatment

The core value of surgery for advanced endometrial cancer is maximizing lesion resection through cytoreductive tumor surgery, thereby creating more favorable conditions for subsequent adjuvant therapy. The decision to pursue surgery for recurrent endometrial cancer depends primarily on whether the recurrence is local or distant/widespread. Furthermore, the extent and timing of surgery must be individualized based on the patient’s molecular subtype and the overall systemic condition.

Surgery in patients with advanced or recurrent endometrial cancer aims to achieve “no gross residual disease” (R0 resection), which is significantly associated with long-term survival (18). Traditionally, surgery for advanced or recurrent disease is performed after a thorough assessment of tumor resectability. Local recurrence is preferably managed with radical approaches, especially in patients who do not initially receive radiotherapy or whose recurrence occurs a considerable time (>12 months) after radiotherapy. The benefits of surgery are limited in patients with extensive peritoneal dissemination or distant organ metastases. Successful surgery is achieved through complete resection, with multiple studies confirming that patients with R0 resection have a significantly superior median overall survival (OS) compared to those with incomplete resection. A phase II trial involving 41 patients with stage III–IV or recurrent endometrial cancer used a “sandwich” protocol (three cycles of docetaxel + carboplatin chemotherapy → surgery → three cycles of chemotherapy + radiotherapy), and reported a 5-year OS rate of 70%; those with no gross residual disease derived even more substantial survival benefit (19). The CEEGOG EX01 trial, which included 230 patients with recurrent endometrial cancer, found that those who achieved R0 resection had a median survival of 113 months compared with 35 months for those who underwent R1/R2 resection (20). The timing of surgery is crucial. For patients with a high tumor burden, in whom direct surgery is challenging, neoadjuvant chemotherapy (NACT) can reduce lesion size and surgical risk. However, NACT may alter the molecular characteristics of tumor cells, potentially impacting subsequent choices for targeted therapy (21).

Molecular classification provides a new basis for surgical decision-making in advanced endometrial cancer. For instance, despite potentially having a high tumor burden, patients with the POLE ultramutated subtype exhibit a high sensitivity to immunotherapy. Overly aggressive surgical resection may increase the risk of complications; therefore, after confirming the molecular subtype via biopsy, prioritizing immunotherapy followed by minimally invasive surgery after tumor reduction should be considered (8). In contrast, tumors in patients with p53abn are highly aggressive. Despite achieving R0 resection, the risk of recurrence remains high, necessitating surgery and systemic chemotherapy to control micro-metastases. Patients with HER2-positive serous endometrial cancer require combined anti-HER2 therapy postoperatively (22). In patients with the MMRd subtype, tumors are highly immunogenic. Although lymph node micro-metastases are present, postoperative immunotherapy may effectively control the disease. Consequently, the scope of lymph node dissection can be appropriately reduced to minimize complications, such as lymphedema (19).However, there is currently a lack of high-level evidence from prospective clinical trials to systematically evaluate the safety and long-term efficacy of the strategy of “reducing the extent of lymphadenectomy,” and further research is still required for confirmation.

### 2.2 Radiotherapy

Radiotherapy is primarily used for postoperative adjuvant treatment, local lesion control, and palliative care in patients with advanced and recurrent endometrial cancer. Its value must be comprehensively evaluated based on the patient’s molecular subtype, residual surgical status, and response to systemic therapy. In patients with gross residual disease (R1/R2 resection) or positive lymph nodes, pelvic external beam radiotherapy (EBRT) combined with vaginal brachytherapy (VBT) can significantly reduce local recurrence. The PORTEC-2 study demonstrated that, for high-risk early-stage patients, the vaginal recurrence rates for VBT and EBRT were similar (2.3% vs. 1.9%, respectively); however, VBT had lower intestinal toxicity (23). This applies to advanced-stage patients, especially those with the ultra-mutated POLE or MMRd molecular subtypes whose tumors exhibit high sensitivity to radiotherapy, which can produce a synergistic effect with immunotherapy (24). MMRd tumors may undergo immunogenic cell death after radiotherapy, thereby enhancing the efficacy of subsequent immunotherapy; thus, sequential immunotherapy following radiotherapy can be considered. Conversely, p53abn tumors showed stronger resistance to radiotherapy, with local control rates from radiotherapy alone of <50%. Therefore, combining radiotherapy with chemotherapy or targeted therapy is necessary to improve its efficacy. Radiotherapy is suitable for patients with local recurrence who are ineligible for or who refuse surgery. Brachytherapy alone can achieve a 5-year local control rate of approximately 70% for vaginal cuff recurrence. For pelvic lymph node recurrence, EBRT combined with chemotherapy (cisplatin sensitization) can increase the local control rate to 60–70% (25, 26). Recently, the use of stereotactic body radiotherapy (SBRT) for oligometastases has gradually increased. Its advantages include a short treatment course (1–5 sessions), minimal damage to normal tissues, and local control rates >80% (27).

### 2.3 Systemic therapy

Systemic therapy serves as the cornerstone of the management of advanced and recurrent endometrial cancer. Chemotherapy forms the backbone, and platinum-based regimens remain a fundamental choice. The rapid development of targeted therapies and immunotherapies has revolutionized the treatment of advanced and recurrent diseases. Particularly, precision medicine guided by molecular classification significantly improves patient outcomes. However, clonal evolution and spatiotemporal heterogeneity of tumors pose major challenges to precision therapy. Differences in molecular subtypes can exist between primary and recurrent tumors and within different regions of the primary tumor itself. One study involving 141 patients with recurrence found subtype conversion in four cases (3.8%), in which two shifted from MMRd to p53abn and two from p53wt to p53abn, suggesting that recurrent lesions may acquire new molecular alterations (28). The clinical implication of this dynamic molecular evolution is that re-evaluation of the molecular profile upon recurrence may alter the treatment strategy.

#### 2.3.1 Chemotherapy

For advanced and recurrent endometrial cancer, the first-line chemotherapy standard of care is combined carboplatin and paclitaxel therapy (TC regimen), yielding an objective response rate (ORR) of 43–62% and a median progression-free survival (PFS) of 5.3–15 months (29). For patients intolerant to paclitaxel, carboplatin combined with liposomal doxorubicin can be used, achieving an ORR of 30–40%, although attention must be paid to adverse effects, such as hand-foot syndrome (30). A retrospective study involving 272 patients with endometrioid adenocarcinoma found that the 5-year OS rate for advanced-stage patients who received postoperative chemotherapy (76.4%) was significantly higher than that for patients who did not (28.6%), whereas the benefit of chemotherapy was not significant for early-stage patients (31). These results suggest that postoperative chemotherapy should be the standard treatment for advanced-stage patients with the molecular p53abn or NSMP subtypes whose tumors carry a high risk of systemic metastasis (2). For patients with the MMRd subtype, chemotherapy combined with radiotherapy is recommended if lymph node metastasis or lymphovascular space invasion (LVSI) is present in the early stages.

#### 2.3.2 Targeted therapy

Targeted therapy and its combination with chemotherapy have become a major research focus in advanced endometrial cancer, primarily concentrating on patients with HER2 positivity, PI3K/AKT/mTOR pathway abnormalities, and sensitivity to PARP inhibitors.

In addition to TP53 mutations, the p53abn subtype of endometrial carcinoma is frequently accompanied by other driver genetic alterations, such as CCNE1 amplification (approximately 30%), PIK3CA mutation (approximately 20%), ERBB2 amplification (approximately 15%-20%), and FGFR2 mutation (approximately 10%). These co-occurring molecular aberrations collectively underpin its biological characteristic of relative insensitivity to conventional chemotherapy (32, 33). Clinical data reveal that for patients with advanced or recurrent p53abn tumors treated with platinum-taxane combination chemotherapy, the objective response rate (ORR) is only 20%-30%, with a median progression-free survival (PFS) of less than 6 months and a median overall survival (OS) of about 12 months, outcomes that are significantly inferior to those observed in the MMRd and POLEmut subtypes (15, 34). Consequently, targeted combination regimens specifically designed for the p53abn subtype have emerged as a focused area of therapeutic development.HER2-positive serous endometrial cancer accounts for approximately 15–20% of advanced cases. These tumors are highly aggressive, and traditional chemotherapy yields an ORR of <30%. Fader et al. demonstrated that the addition of trastuzumab to carboplatin and paclitaxel chemotherapy regimens significantly improved PFS and OS in patients with HER2-positive serous carcinoma (35, 36). Research on novel anti-HER2 agents is ongoing, with preliminary results indicating their efficacy even in patients resistant to trastuzumab. Early studies on antibody-drug conjugates (ADCs), such as HER2-targeting T-DXd, showed promising prospects in patients with HER2-positive recurrence, with ORRs reaching >40% (8). A phase II study showed that sacituzumab govitecan achieved an ORR of 25% and a median PFS of 5.5 months in advanced-stage patients previously treated with at least two lines of therapy (37). This drug is suitable for patients with HER2-low expression or triple-negative profiles and offers a new therapeutic option. For patients with high VEGF expression, chemotherapy combined with bevacizumab can significantly prolong PFS, although vascular toxicities, such as hypertension and proteinuria, require monitoring. PARP inhibitors are suitable for patients with BRCA mutations or homologous recombination deficiency (HRD), with an ORR of 30–40% and a median PFS of 9.2 months (38). They exhibit potential efficacy in p53abn patients carrying BRCA1/2 mutations or those with high genomic instability scores (39, 40). An ongoing p53abn-RED clinical trial (NCT05255653) is evaluating the efficacy of olaparib combined with chemoradiotherapy in p53abn patients (41). PI3K/AKT/mTOR pathway inhibitors combined with endocrine therapy can be used for hormone receptor-positive advanced cancer patients, with an ORR of 20–30% and a median PFS of 6–8 months (29).

Recent advances in the nuclear export inhibitor selinexor have provided a new direction for maintenance therapy in p53 wild-type endometrial cancer. A subgroup analysis from the ENGOT-EN5/GOG-3055/SIENDO study showed that maintenance therapy with single-agent selinexor significantly prolonged progression-free survival in p53 wild-type patients, with a median PFS of 27.4 months, which was vastly superior to 5.2 months in the placebo group (42). The ongoing ENGOT-EN20/GOG-3083/XPORT-EC-042 phase III clinical trial (NCT05611931) will further validate the value of selinexor as maintenance therapy in p53 wild-type advanced or recurrent endometrial cancer (43).

#### 2.3.3 Immunotherapy

**Figure 2:**
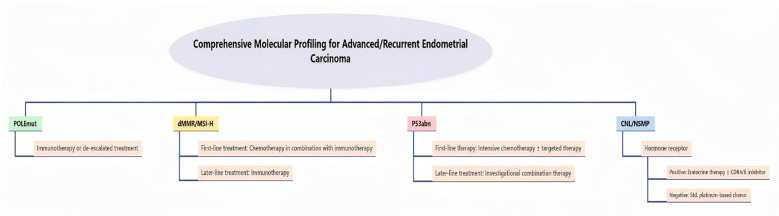
Molecular classification-guided treatment pathway for advanced/recurrent endometrial cancer.

Microsatellite instability (MSI) or mismatch repair deficiency (dMMR) status of tumors has become a primary indicator of immunotherapy owing to: (i) MMRd tumors exhibit a tumor mutational burden (TMB) ranging from 100 to 1000 mutations per megabase (mut/Mb), which is significantly higher than that in non-MMRd tumors (typically <10 mut/Mb) (15); (ii) The density of tumor-infiltrating lymphocytes (TILs), particularly cytotoxic CD8^+^ T cells and helper CD4^+^ T cells, is markedly increased in MMRd tumors, concurrently accompanied by a significant upregulation in the expression levels of the PD-1/PD-L1 pathway. The RUBY trial, investigating first-line immunotherapy combined with chemotherapy (dostarlimab + carboplatin/paclitaxel), as the first Phase III clinical trial to validate the benefit of this combination in the first-line treatment of advanced and recurrent endometrial cancer,showed that PFS was significantly prolonged in patients with dMMR (median not reached vs. 7.7 months) (44). Safety analysis revealed that the incidence of treatment-related adverse events (TRAEs) was similar between the combination therapy group and the placebo group (97.9% vs. 98.8%). However, the incidence of grade 3–4 TRAEs was slightly higher in the combination group (50.2% vs. 46.7%). The rate of treatment discontinuation due to adverse events (AEs) was 15.8% in the combination group, comparable to 14.2% in the placebo group, suggesting an acceptable tolerability profile for first-line immunotherapy combined with chemotherapy (45). The results of the RUBY trial directly contributed to the update of the NCCN guidelines, which now recommend dostarlimab in combination with carboplatin-paclitaxel as a preferred first-line regimen for advanced and recurrent endometrial cancer, regardless of MMR status (46).The KEYNOTE-158 study demonstrated that pembrolizumab monotherapy for dMMR/MSI-H advanced endometrial cancer achieved an ORR of 47% and a median OS of 24.3 months. In the GARNET study, dostarlimab monotherapy in patients with advanced dMMR yielded an ORR of 42.3% and the median duration of response (DOR) had not been reached (47). These results indicate that for patients with advanced dMMR/MSI-H, immunotherapy monotherapy can be a standard option, especially after chemotherapy failure (48), and can serve as a first-line “chemotherapy-free” regimen. For patients with proficient mismatch repair (pMMR) or microsatellite stable (MSS) tumors, the efficacy of immunotherapy monotherapy is limited and requires combined targeted therapy to improve outcomes. The phase III KEYNOTE-775 trial enrolled 827 patients with advanced or recurrent endometrial cancer who had progressed on prior platinum-based chemotherapy, randomizing them to receive either pembrolizumab plus lenvatinib or investigator’s choice of chemotherapy, showed that pembrolizumab combined with lenvatinib in patients with advanced pMMR resulted in an ORR of 36% and a median PFS of 7.4 months, which were significantly better than those in the chemotherapy group (3.8 months) (49). Regarding safety, the incidence of any-grade adverse events (AEs) was similar between the combination therapy group and the chemotherapy group. However, the rate of grade 3–4 AEs was substantially higher in the combination group (88.9% vs. 72.7%) (50). This was particularly pronounced in elderly patients (≥70 years), where the incidence of grade 3–4 AEs reached 60.0%, underscoring the need for more cautious dose optimization in this population (51). This combination regimen has been approved by the U.S. FDA for the treatment of advanced or recurrent endometrial cancer following prior platinum-based chemotherapy, regardless of MMR status, and represents a critical option, especially for patients who are intolerant to chemotherapy (50). It not only addresses a significant therapeutic gap for patients with microsatellite-stable/proficient mismatch repair (MSS/pMMR) tumors but also firmly establishes “immunotherapy plus anti-angiogenic therapy” as a standard second-line treatment (52). Real-world data further validated the effectiveness of this combination immunotherapy. A real-world study involving 326 advanced-stage patients showed that the median time to the following treatment after pembrolizumab plus lenvatinib was 11.2 months, which was significantly longer than that in the chemotherapy group (7.7 months) (53). Another case report described a patient with advanced pMMR who achieved 5-year PFS after treatment with pembrolizumab and lenvatinib (54). These findings suggest that the efficacy of the immunotherapy-targeted therapy combination in the real world aligns with the clinical trial results. The NSMP subtype accounts for the highest proportion of patients (approximately 40–50%). Among them, patients with ARID1A mutations have a significantly increased risk of recurrence (hazard ratio [HR]=3.96, 95% confidence interval [CI]; 1.41–11.15) and may require more aggressive adjuvant therapy (26, 55). Conversely, patients with hormone receptor-positive NSMP can benefit from endocrine therapy, especially elderly patients or those with poor performance status. Commonly used drugs include megestrol acetate, tamoxifen, and aromatase inhibitors, with an ORR of approximately 20–30% and a median PFS of approximately 3–6 months (8, 56). The combination of CDK4/6 inhibitors and endocrine therapy has shown promising efficacy and tolerability in hormone receptor-positive endometrial cancer, potentially becoming an important component of first-line chemotherapy-free strategies (57, 58). Furthermore, a study of grade 3 endometrioid carcinoma showed that patients with the NSMP subtype harboring PIK3CA or PTEN mutations were sensitive to PI3K/AKT/mTOR pathway inhibitors, providing a rationale for targeted therapy in this group (**Figure 2**) (40, 59).

### 2.4 Mechanisms of immunotherapy resistance and strategies to overcome them

The efficacy of immunotherapy is suboptimal in patients with pMMR-type disease, making the elucidation of resistance mechanisms a core challenge in current research. Existing studies indicate that immunotherapy resistance can be categorized into primary and acquired resistance involving multiple factors, such as tumor cell-intrinsic factors, abnormalities in the immune microenvironment, and host factors. At the tumor cell level, common causes of resistance include loss of HLA molecule expression, defects in the antigen presentation machinery, aberrations in the IFN-γ signaling pathway, and downregulation of PD-L1 expression (60, 61). Aberrant remodeling of the immune microenvironment is significant in immunotherapy resistance. Immunosuppressive cells within the tumor microenvironment, such as regulatory T cells, myeloid-derived suppressor cells, immune checkpoint molecules, and metabolic reprogramming, collectively form immunosuppressive barriers that impede the infiltration and function of effector T cells (60, 61). Furthermore, the physical barrier formed by extracellular matrix components secreted by cancer-associated fibroblasts further restricts the depth of immune cell infiltration (62). Therefore, combination strategies, such as combining immune checkpoint inhibitors with antiangiogenic agents, chemotherapy, or radiotherapy, aim to remodel the immune microenvironment and enhance the immune response.

**Figure 3:**
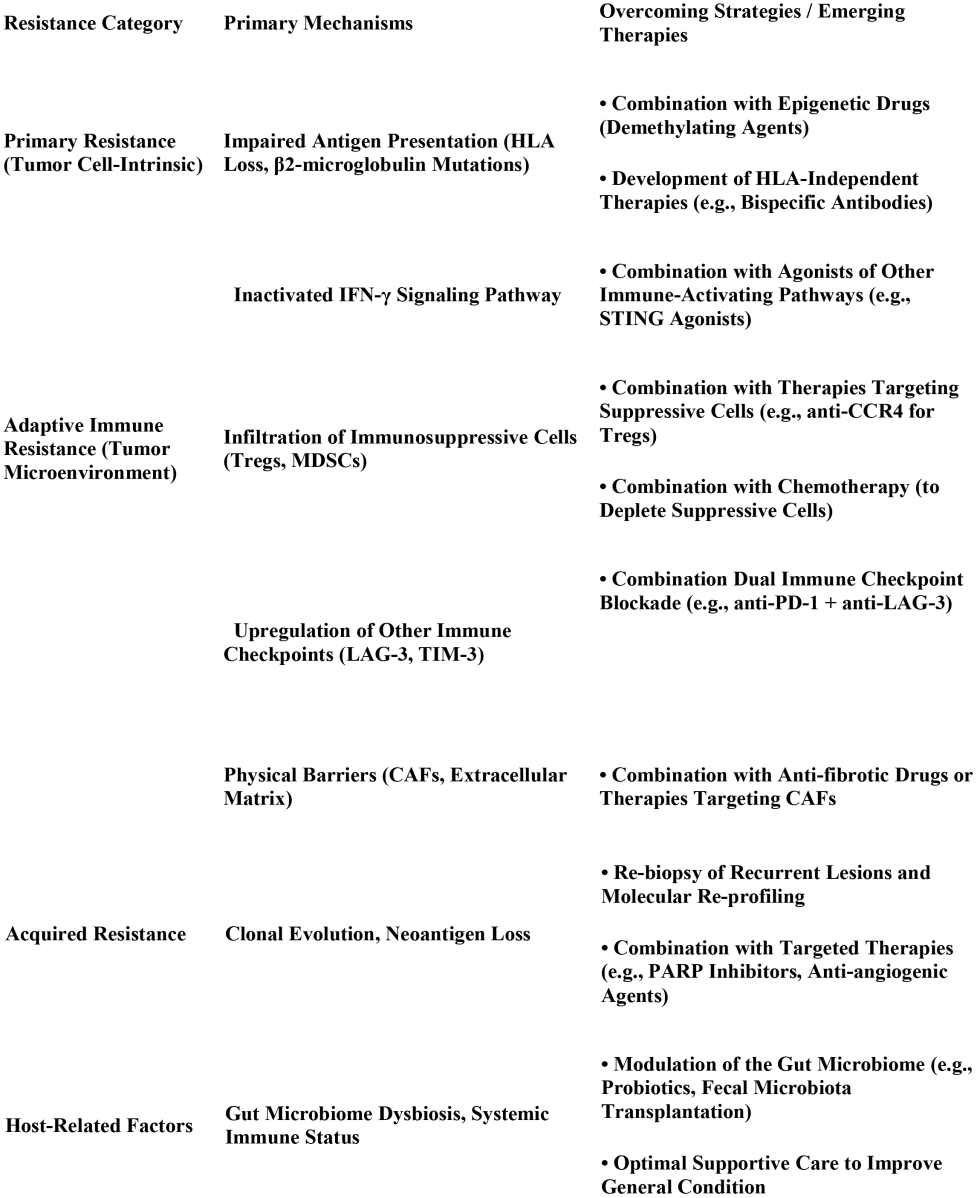
Endometrial cancer immunotherapy resistance: mechanisms and management strategies.

Combination strategies for overcoming resistance to immunotherapy are continuously being developed. Combined chemotherapy and immunotherapy has become the first-line standard of care for patients with advanced endometrial cancer. Previously, the RUBY trial demonstrated that this combination significantly prolonged PFS in patients with dMMR. Similarly, the NRG GY018 study is a phase II clinical study designed to evaluate the efficacy and safety of three targeted combination regimens—pembrolizumab plus lenvatinib, pembrolizumab plus olaparib, and lenvatinib plus olaparib—in patients with advanced or recurrent endometrial cancer who have received at least one prior line of platinum-based chemotherapy, showed that pembrolizumab combined with carboplatin/paclitaxel significantly extended PFS compared to chemotherapy alone, regardless of the MMR status (63, 64). Building on the findings of KEYNOTE-775, the NRG GY018 trial not only corroborated the efficacy of the pembrolizumab-plus-lenvatinib regimen in the second-line setting but also explored novel combination strategies involving PARP inhibitors paired with either immunotherapy or anti-angiogenic therapy. These explorations provide a new therapeutic avenue for patients with homologous recombination deficiency (HRD)-positive tumors. Furthermore, the differential efficacy observed among the three distinct combination regimens underscores the necessity of biomarker-guided selection to identify the optimal treatment strategy for individual patients (65).The combination of dual immune checkpoint inhibitors further improves response rates and duration of response in patients with dMMR. Although dostarlimab monotherapy achieved an ORR of 49% in dMMR advanced endometrial cancer, combining it with a CTLA-4 inhibitor may further enhance its efficacy (47, 66). Additionally, a combination of immunotherapy and PARP inhibitors (as observed in the DUO-E study) demonstrated synergistic effects in advanced endometrial cancer, particularly in HRD-positive patients, providing a rationale for exploring “chemotherapy-free” regimens (58, 63). The development of novel immunotherapeutic agents has provided new tools to combat drug resistance. Besides traditional PD-1/PD-L1 inhibitors, clinical trials are evaluating new immune checkpoint inhibitors, T-cell agonists, and bispecific antibodies. The CD276-targeted bispecific antibody CC-3, which simultaneously binds to CD276 on tumor cells and CD3 on T cells, recruits T cells to the tumor site and activates their killing function, demonstrating potent cytotoxicity against endometrial cancer cell lines *in vitro* (67). Furthermore, clinical trials of adoptive cell therapies targeting tumor-specific antigens (e.g., NY-ESO-1) are underway and offer potential treatment options for advanced treatment-resistant patients (**Figure 3**).

## 3 Future perspectives

Molecular classification is the cornerstone of current treatment decision-making for endometrial cancer. The TCGA and ProMisE classification systems have clearly categorized patients into POLE-mutated, dMMR/MSI-H, p53abn, and NSMP subtypes, each with a distinctly different prognosis and treatment response (7, 8). Patients with the POLE-mutated subtype have excellent prognosis and may be suitable for treatment de-escalation. Patients with the dMMR/MSI-H subtype were highly sensitive to immunotherapy, achieving an ORR of 51.9%, which was significantly higher than the 16.1% observed in the pMMR population. In contrast, patients with the p53abn subtype have the poorest prognosis and require more intensive treatment strategies (52). Real-world studies have indicated that only approximately one-third of advanced-stage patients undergo MSI/MMR testing, which severely limits the implementation of precision therapies (68, 69). Therefore, clinical practice should vigorously promote molecular classification testing, including IHC for MMR protein expression or NGS for MSI status, to ensure that every patient receives an individualized treatment plan based on subtype (55, 70).

Discrepancies exist between real-world effectiveness data and clinical trial results. These differences can be primarily attributed to factors such as the heterogeneity of patient populations (e.g., age, performance status, comorbidities, and molecular tumor subtypes), the diversity and flexibility of treatment regimens, variations in healthcare resources and treatment standards, as well as real-world management practices including treatment interruptions and dose adjustments. For instance, in a UK real-world study, the median patient age was 68.5 years, with 22.9% having an ECOG PS of 2, whereas in the KEYNOTE-775 trial, the median age was 65 years, and patients with PS ≥ 2 were excluded. The incidence of grade 3–4 adverse reactions associated with lenvatinib plus pembrolizumab was 55.7% in the real-world study, notably lower than the rate reported in KEYNOTE-775. This difference likely stems from more proactive dose modifications or treatment interruptions by physicians in real-world clinical practice. Real-world data serve to both complement and refine conclusions drawn from clinical trials. They first validate the external validity of trial findings. Second, they provide insights into patient populations that are typically underrepresented or excluded from clinical trials. Ultimately, this integration aims to optimize treatment decision-making. Therefore, future efforts should strengthen the synergy between real-world research and clinical trials. By amalgamating data from both sources, we can furnish a more robust evidence base for the personalized treatment of advanced and recurrent endometrial cancer.

Targeted modulation of the TME is currently a research hotspot. Cancer-associated fibroblasts (CAFs), a major component of the TME, promote endometrial cancer cell proliferation and drug resistance via cytokine secretion. Optimized organoid models, such as coculture systems of CAFs with tumor cells, simulate the *in vivo* TME and serve as platforms for drug sensitivity screening, thereby supporting the development of individualized treatment strategies (62). Regarding targeted therapeutic strategies against the immunosuppressive microenvironment, the CD276 (B7-H3)-targeting bispecific antibody CC-3 has entered phase I clinical trials (67). These investigations provide novel solutions to enhance the efficacy of immunotherapy and overcome immune resistance.

The microbiome has emerged as a potential modulator of tumor immune response, garnering increasing attention in the field of advanced and recurrent endometrial cancer. MSI-H/dMMR subtypes, owing to their high tumor mutational burden and enhanced immunogenicity, may harbor a tumor microenvironment enriched with immunostimulatory microbiota, whereas the p53abn subtype may exhibit a microbiota profile skewed toward an immunosuppressive phenotype (13). Clinical investigations are currently underway to explore the relationship between the microbiome and immunotherapy response in endometrial cancer. In a prospective study (NCT04163289), pretreatment gut microbiome profiling via 16S rRNA sequencing revealed that patients with a higher abundance of Prevotella achieved a significantly higher objective response rate (ORR) to pembrolizumab plus lenvatinib compared to those with low microbial diversity (62% vs. 28%, P = 0.03) (71). A separate retrospective analysis of fecal samples from the KEYNOTE-775 trial demonstrated that, in MSI-H patients, higher intestinal abundance of Faecalibacterium prausnitzii was associated with prolonged progression-free survival (HR = 0.45, 95% CI 0.23–0.88), whereas no such correlation was observed in p53abn patients (72). These preliminary findings suggest that the microbiome may serve as a novel biomarker for predicting immunotherapy response, though further validation is required to elucidate its underlying mechanisms and clinical applicability.

Advanced and recurrent endometrial cancer treatment has entered the era of precision medicine. Molecular classifications and immunotherapy have significantly improved patient outcomes. In clinical practice, it is essential to adhere to molecular classification guidelines, prioritize the selection of optimal treatment regimens, and simultaneously emphasize the management of complications and quality of life. Future research should focus on optimizing the molecular classification, overcoming immunotherapy resistance, and conducting real-world studies to further enhance treatment efficacy and achieve individualized therapy. Interdisciplinary collaboration, international data sharing, and patient engagement are crucial forces that drive progress in this field.
